# The diagnostic yield of CGH and WES in neurodevelopmental disorders

**DOI:** 10.3389/fped.2023.1133789

**Published:** 2023-03-01

**Authors:** Raniah S. Alotibi, Naif S. Sannan, Mariam AlEissa, Marwh G. Aldriwesh, Abeer Al Tuwaijri, Maaged A. Akiel, Mashael Almutairi, Alhanouf Alsamer, Nouf Altharawi, Ghadah Aljawfan, Badi Alotiabi, Mohammed A. AlBlawi, Ahmed Alfares

**Affiliations:** ^1^ Department of Clinical Laboratory Sciences, College of Applied Medical Sciences, King Saud bin Abdulaziz University for Health Sciences (KSAU-HS), Riyadh, Saudi Arabia; ^2^ King Abdullah International Medical Research Center (KAIMRC), Riyadh, Saudi Arabia; ^3^ Department of Clinical Laboratory Sciences, College of Applied Medical Sciences, King Saud bin Abdulaziz University for Health Sciences (KSAU-HS), Jeddah, Saudi Arabia; ^4^ Department of Molecular Genetics, Public Health Laboratory, Public Health Authority, Riyadh, Saudi Arabia; ^5^ College of Medicine, Alfaisal University, Riyadh, Saudi Arabia; ^6^ Medical Genomics Research Department, King Abdullah International Medical Research Center (KAIMRC), King Saud Bin Abdulaziz University for Health Sciences, Ministry of National Guard Health Affairs (MNGH), Riyadh, Saudi Arabia; ^7^ Department of Pathology and Laboratory Medicine, King Abdulaziz Medical City, Riyadh, Saudi Arabia; ^8^ College of Medicine, King Saud bin Abdulaziz University for Health Sciences (KSAU-HS), Riyadh, Saudi Arabia; ^9^ Center for Genomic Medicine, King Faisal Specialist Hospital & Research Centre, Riyadh, Saudi Arabia; ^10^ Department of Pediatrics, College of Medicine, Qassim University, Qassim, Saudi Arabia

**Keywords:** array-based comparative hybridization (Array-CGH), whole exome sequencing (WES), copy number variations (CNVs), single-nucleotide variants (SNVs), neurodevelopmental disorders (NDDs)

## Abstract

**Background:**

Neurodevelopmental disorders are a group of conditions characterized by developmental delays leading to abnormal brain functions. The methods of diagnosis and treatment of these conditions are complicated, and their treatment involves a combination of various forms of therapy. In recent years, the development of high-resolution technologies has played an important role in revealing the microdeletions, microduplications, and single-nucleotide variants of the chromosomes and how they are linked to the development of neurodevelopmental disorders. The wide implementation and application of molecular methodologies have started to shed light on the functional importance of using the appropriate methods in detecting these genetic variations that are categorized as either pathogenic or benign. The study aimed to compare the diagnostic yield of comparative hybridization (CGH) and whole exome sequencing (WES) in neurodevelopmental disorders among children attending the King Abdullah Specialist Children Hospital, Riyadh, Saudi Arabia.

**Methods:**

A retrospective study was conducted between 2015 and 2018 on 105 patients diagnosed with neurodevelopmental disorders through array-based CGH (Array-CGH) and WES.

**Results:**

In a sample of 105 patients, 16% was the hit rate of copy number variations (CNVs). WES was requested for CNV-negative patients (*n* = 79), of which 30% was the hit rate of pathogenic or likely pathogenic single-nucleotide variants. There was a difference in the diagnostic yield between CGH (16%) and WES (30%).

**Conclusion:**

WES was a better approach than Array-CGH to detect various DNA mutations or variants. Our findings could guide clinicians, researchers, and testing laboratories select the most cost-effective and appropriate approach for diagnosing their patients.

## 1. Introduction

Neurodevelopmental disorders (NDDs) are impairments of the growth and development of the brain and/or central nervous system leading to delays in acquisition of skills during human development. The disorders affect various developmental areas including social, cognition, language, and motor development domains (1). Numerous NDDs can affect children and adolescents of all ages from 1 month old to adolescents and young adults of 21 years (2). NDDs include attention-deficit/hyperactivity disorder (ADHD), learning disabilities, autism spectrum disorder (ASD), cerebral palsy, intellectual disability, and other disorders (3–5). Children who are affected by these disorders are unable to perform various neurological functions, such as learning, storing memory, developing appropriate speech and/or language, behavior changes, and motor skills. Some of the NDD conditions change over time as the child grows, while others may persist and are considered permanent (6, 7). The methods of diagnosis and treatment of NDD conditions can be complicated, and their treatment involves a combination of various forms of therapy, which may include the use of physician-administered drugs and other home-based and school-based activities (8–11). Fifteen percent of children in Saudi Arabia aged between 1 month and up to 21 years were affected by NDDs including autism, intellectual disability, ADHD, learning disabilities, and problems with speech development and behavior (12).

In recent years, the development of high-resolution technology has played an important role in revealing the microdeletions, microduplications, and single-nucleotide variations of DNA sequences and how they are linked to the development of NDDs (1). Array-based comparative hybridization (Array-CGH) is one of the developed technologies, which has enhanced knowledge regarding these deleterious mutations that occur in human chromosomes (3, 4). The microduplications and/or microdeletions of specific regions within the human chromosome, whose size ranges from a few hundred base pairs to over a million bases, are referred to as copy number variations (CNVs) (13). They are essential and play a crucial role in phenotypic diversity and evolution of the human genome (14, 15). Most copy number variations have no harm to individuals involved; however, some are associated with diseases that affect human beings, including several NDDs such as autism spectrum disorder, ADHD, and intellectual disability (16–21).

A variation in a single nucleotide is referred to as single-nucleotide variants (SNVs), which are increasingly detected using technological advancement in molecular methodology and extensive utilization of whole exome sequencing (WES), which generates massive amounts of genomic variant information. Selecting the most effective method and interpreting the results presents a major challenge to medical practitioners to identify which variations drive disease or contribute to phenotypic traits (19). However, more research is necessary to elucidate the various mechanisms of these genetic variations and how they influence NDDs. This retrospective research aims to compare the diagnostic yield of CGH and WES and determine the most effective method of identification of genetic variations in NDDs among children attending King Abdullah Specialist Children Hospital (KASCH) in Saudi Arabia.

## 2. Methods

### 2.1. Study population

The study was conducted at the Molecular and Diagnostic Central Laboratory, KASCH, Riyadh, Saudi Arabia. A retrospective study on 105 patients diagnosed with NDDs between 2015 and 2018.

### 2.2. Inclusion criteria

The participants had to be aged between 1 month and 19 years and have unexplained NDDs, which could include developmental delay disorder, epilepsy, intellectual disability, learning disorder, and/or intellectual disability. Their DNA profiles were investigated through Array-CGH and/or WES.

### 2.3. Exclusion criteria

There were no specific exclusion criteria.

### 2.4. Array-based comparative genomic hybridization

Whole genomic array-based comparative genomic hybridization (aCGH) and genotype analyses are performed on a custom-designed oligonucleotide microarray (GenomDx v5). The array design is based on human genome build GRCh37/UCSC hg19, and results are reported according to the current The International System for Human Cytogenomic Nomenclature ISCN guidelines. The array contains approximately 118,000 probes that provide copy number data and 66,000 probes that generate genotype information through analysis of SNPs. Reported boundaries correspond to deviating probes, which are dependent on array design and have the inherent limitation of not reflecting exact aberration breakpoints. For testing performed on blood samples, the array detects copy number changes of >200 kb, on average, across the entire unique sequence of the human genome and between 500 and 15 kb in more than 200 targeted regions. The array also detects >5 Mb regions of homozygosity (ROH). ROH is reported if there is at least one region >10 Mb, or two regions each >8 Mb, suggesting identity by descent. The possibility of uniparental disomy (UPD) is reported when there is a single terminal ROH >10 Mb or interstitial ROH >20 Mb in the absence of other reportable ROH.

### 2.5. Whole exome sequencing

Total genomic DNA was extracted from biological sample using a spin column method. DNA quality and quantity were assessed using electronic methods; after assessment of DNA quality, qualified genomic DNA samples were randomly fragmented using noncontact, isothermal sonochemistry processing and purified with Solid Phase Reversible Immobilisation (SPRI) beads. Then, DNA fragments were end repaired and sequencing adapters were ligated to both ends of the resulting fragments. Prepared DNA-Adapter libraries were size-selected with SPRI beads to ensure optimal template size and then amplified by ligation-mediated PCR (LM-PCR). The amplified sequencing library was again purified using SPRI beads and hybridization—the capture method was applied for enrichment of the whole exome and selected noncoding regions. The enriched sequencing library was amplified by LM-PCR and purified using SPRI beads. The completed sequencing library that passed quality control was sequenced using Illumina sequencing system (The NextSeq 550). Paired-end sequencing (150 by 150 bases) was performed to yield the required number of reads (100M). Sequencing-derived raw image files were processed using a base-calling software (Illumina), and the sequence data were transformed to FASTQ format. The bioinformatics analysis began with quality control of raw sequence read. Clean sequence reads of each sample was mapped to the human reference genome (GRCh37/hg19). Burrows–Wheeler Aligner (BWA-MEM) software was used to read the alignment. Duplicate read marking, local realignment around indels, base quality score recalibration, and variant calling were performed using GATK algorithms (Sentieon). The sequencing depth and coverage for each individual were calculated based on the alignments. Each exome batch was subjected to thorough quality control measures, after which raw sequence reads were transformed into variants by a proprietary bioinformatics pipeline. Samples tested with WES required ∼90× depth of coverage, and the minimum coverage for any variant to be considered is 20×. The configuration of the pipeline was based on the sequencing systems and types of the kits.

The classification of variants as pathogenic/likely pathogenic (P/LP), a variant of uncertain significance (VOUS), or benign was predicted based on the American College of Medical Genetics and Genomics (ACMGG) scoring system (22).

Detailed clinical information of NDD according to the Human Phenotype Ontology (HPO) format is provided below.

**NDD** [HP:0012758] refers to delays in the maturation of the brain and central nervous system; infants and young children with NDD may experience delays in the development of one or more skills including gross motor abilities, fine-motor coordination, language abilities, and ability to solve increasingly complex problems.

**Epilepsy** [HP:0001250] is an intermittent abnormality of nervous system physiology characterized by a transient occurrence of signs and/or symptoms due to abnormal excessive or synchronous neuronal activity in the brain.

**Intellectual disability** [HP:0001249] is subnormal intellectual functioning which originates during the developmental period. Intellectual disability, previously referred to as mental retardation, has been defined as an IQ score below 70.

**Developmental delay** [HP:0001263] is a delay in the achievement of motor or mental milestones in the domains of development of a child, including motor skills, speech and language, cognitive skills, and social and emotional skills. This term should only be used to describe children younger than 5 years of age.

**Learning disability** [HP:0001328] refers to impairment of certain skills such as reading or writing, coordination, self-control, or attention that interfere with the ability to learn. The impairment is not related to a global deficiency of intelligence.

In order to obtain a diagnosis for NDD cases, several factors are incorporated to reach one or few variants. These factors include (1) the patient clinical phenotypes, (2) mode of inheritance, and (3) allele frequency in a population database. Several tools were used, including VarSeq software from GoldenHelix (http://www.goldenhelix.com/) for filtration process, Alamut® Visual (http://www.interactive-biosoftware.com/alamut visual/), BaseSpace Variant Interpreter (illumina.com), VarSome The Human Genomics Community, Decipher database: (https://www.deciphergenomics.org/ddd/research-variants), Gnomad database (https://gnomad.broadinstitute.org/), and The Phenomizer—Clinical Diagnostics with Similarity Searches in Ontologies (charite.de).

### 2.6. Demographic data

The demographic information of the 105 participants in the study included both male and female children from Saudi Arabia. The data are summarized in Table 1.

**Table 1:** Demographic data of study participants.

Demographic data	*N*
**Age groups**	
1 month–2 years	63
3–6 years	31
7–11 years	8
12–19 years	3
**Gender**	
Male	72 (68%)
Female	33 (31%)

### 2.7. Data collection

Patients’ clinical data were retrospectively extracted from the patients’ clinical records. Data included family history, neuropsychiatric evaluation, and CNV-related information, such as deletions and/or duplications of chromosomes, multiple rearrangements, SNVs, and the presence of interrupted genes. The details were obtained by a thorough review of clinical reports present at KASCH health records. Found DNA variations were grouped into P, LP, and VOUS based on the ACMGG scoring system (22).

### 2.8. Data analysis

We used SPSS (Statistical Package for the Social Sciences v21.00) for analyzing the percentage (frequency) and describing the categorical variable.

### 2.9. Ethical considerations

The study was reviewed and approved by the Institutional Review Board Office at King Abdullah International Medical Research Center (KAIMRC) in Riyadh, Saudi Arabia (Protocol Approval Number SP 19/161/R). All patients have been consented to be enrolled in this study; a written consent form was obtained from all parents’ patients.

### 2.10. Data access

The authors declare that the data supporting the findings of this study is available within the paper and its supplementary material.

## 3. Results

**Figure 1:**
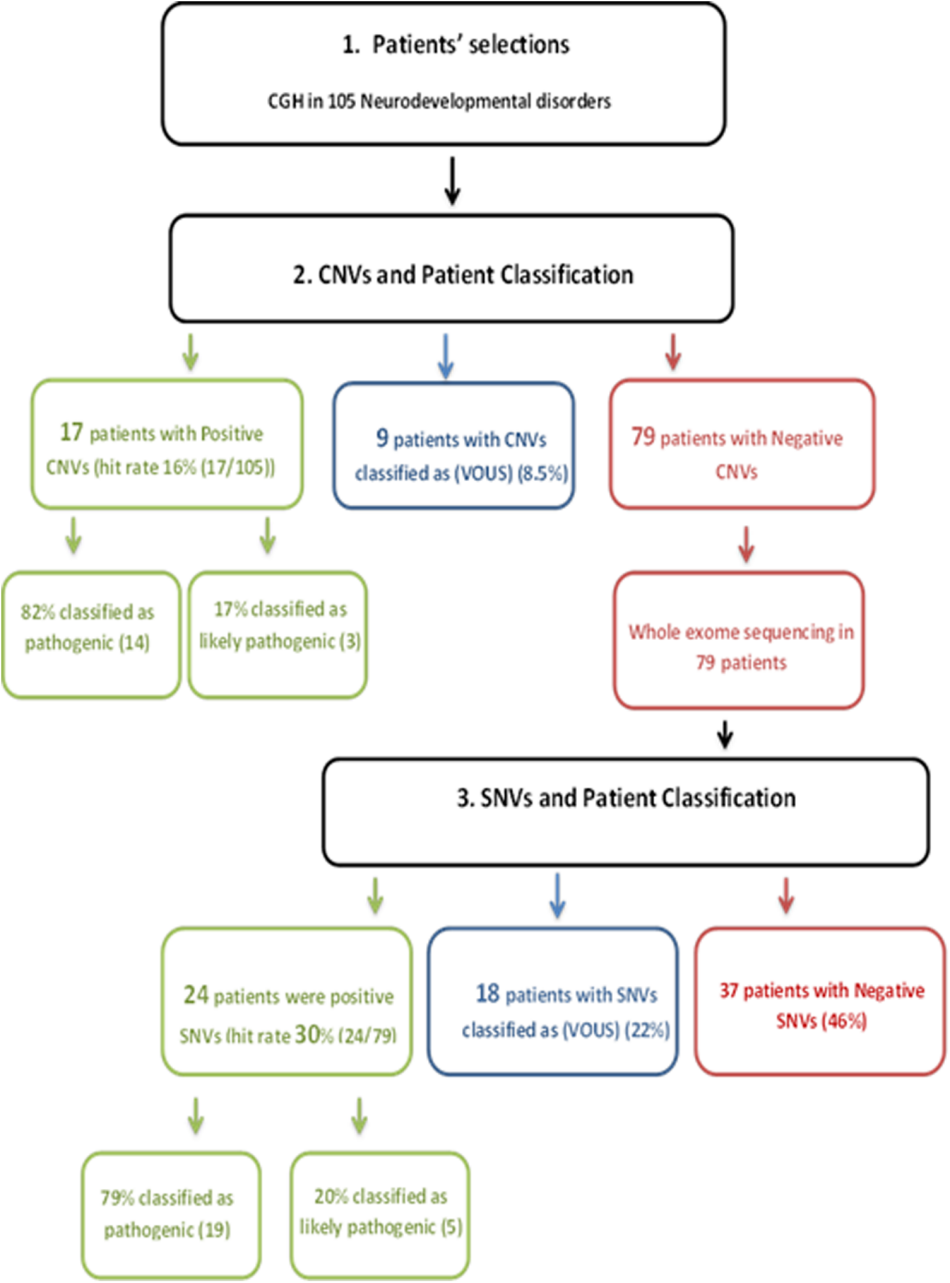
Flowchart of the process followed in this study: 105 patients with age range from 1 month to 19 years diagnosed with specialist investigated through CGH and/or WES, 16% was the hit rate of CNVs. 82% of positive CNVs were classified as pathogenic and 17% were classified as likely pathogenic; 79 patients out of 105 were CNV negative and WES was subsequently requested for those patients. Twenty-four patients out of 79 were positive as pathogenic or likely pathogenic SNVs. The hit rate of SNVs was 30%; 79% were classified as pathogenic and 20% as likely pathogenic. A variant of uncertain significance was reported in 27 patients; 9 patients were classified to have CNVs and the rest 18 patients have SNVs. CGH, comparative hybridization; WES, whole exome sequencing; CNVs, copy number variations; SNVs, single-nucleotide variants.

**Figure 2:**
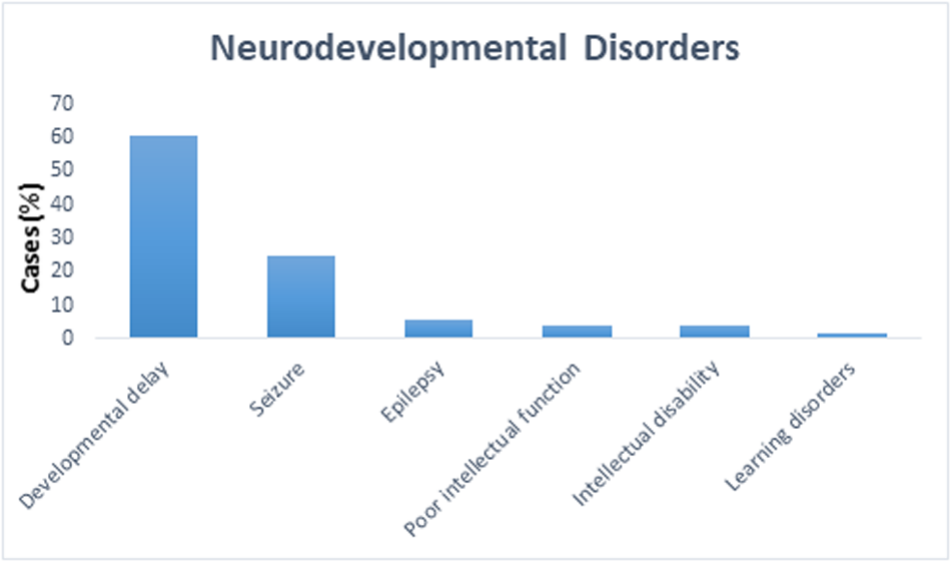
The percentage of different neurodevelopmental disorders among all 105 cases. Developmental delay was the most frequently observed formed 60%, while 5% of patients had epilepsy, 4% had intellectual disability, and 1% had learning disorders.

We present a retrospective study on 105 patients with age range from 1 month to 19 years diagnosed with a specialist investigated through CGH and/or WES between 2015 and 2018. Of the total sample of 105 patients enrolled (Figure 1), 16% was the hit rate of CNVs; 82% of positive CNVs were classified as pathogenic and 17% were classified as likely pathogenic. Moreover, 79 out of 105 patients were CNV negative and WES was subsequently requested for those patients. Out of 79 patients, 24 were positive as pathogenic or likely pathogenic SNVs. The hit rate of SNVs was 30%, 79% were classified as pathogenic and 20% as likely pathogenic. A variant of uncertain significance was reported in 27 patients, 9 patients were classified to have CNVs, and the rest 18 patients have SNVs. Developmental delay was the most frequent NDD observed in 60% of patients, while 5% of patients were affected by epilepsy, 4% by intellectual disability, and 1% had a learning disorder (Figure 2). A hemizygous novel variant was detected by whole exome sequencing in the *FLNA* gene (*C.7906G > A*), which is implicated in developmental delay. Several genes and SNVs were involved in development delays, such as *VPS13B*, *RNASEH2A*, and *WWOX* (Table 2). Table 3 shows CNVs variants found in NDDs.

**Table 2:** SNV variants found in NNDs.

SNVs	Zygosity	ACMG classification	Gene-OMIM phenotype
*VPS13B (NM_152564.5):c.1219C > T (p.Gln407Ter)*	*Homozygous*	Pathogenic	Cohen syndrome
*RNASEH2A(NM_006397.3):c.557G > A (p.Arg186Gln)*	*Homozygous*	Pathogenic	Aicardi–Goutieres syndrome-4
*WWOX (NM_016373.4):c.606-1G > A*	*Homozygous*	Pathogenic	Developmental and epileptic encephalopathy-28, esophageal squamous cell carcinoma, somatic, spinocerebellar ataxia, autosomal recessive-12
*PAH (NM_001354304.2):c.1139C > T* *(p.Thr380Met)*	*Homozygous*	Pathogenic	Phenylketonuria, hyperphenylalaninemia
*PTPN11 (NM_001330437.2):c.1519G > A* *(p.Gly507Arg)*	*Heterozygous*	Pathogenic	LEOPARD syndrome-1, leukemia, juvenile myelomonocytic, metachondromatosis, Noonan syndrome-1
*SPAST (NM_014946.4):c.1496G > A* *(p.Arg499His)*	*Heterozygous*	Pathogenic	Spastic paraplegia-4
*MYT1l (NM_001329851.2):c.1585G > A* *(p.Gly529Arg)*	*Heterozygous*	Pathogenic	Intellectual developmental disorder-39
*TBCD (NM_005993.5):c.1661C > T* *(p.Ala554Val)*	*Homozygous*	Pathogenic	Encephalopathy, progressive, early-onset, with brain atrophy and thin corpus callosum
*SLC19A3 (NM_025243.4):c.1264A > G* *(p.Thr422Ala)*	*Heterozygous*	Pathogenic	Thiamine metabolism dysfunction syndrome-2
*TCF12 (NM_001322164.2):c.493G > T* *(p.Gly165Trp)*	*Heterozygous*	Pathogenic	Craniosynostosis-3, hypogonadotropic hypogonadism-26 with or without anosmia
*QARS1 (NM_005051.3):c.1058G > T* *(p.Gly353Val)*	*Homozygous*	Pathogenic	Microcephaly, progressive, seizures, and cerebral and cerebellar atrophy
*MCOLN1 (NM_020533.3):c.1336G > A* *(p.Val446Met)*	*Homozygous*	Pathogenic	Mucolipidosis-IV
*BRAF (NM_001378474.1):c.1729C > A* *(p.Leu577Ile)*	*Heterozygous*	Pathogenic	Adenocarcinoma of lung, cardiofaciocutaneous syndrome, Colorectal cancer, LEOPARD syndrome-3, Melanoma, Nonsmall cell lung cancer, Noonan syndrome-7
*ZBTB18 (NM_205768.3):c.139°C > T* *(p.Arg464Cys)*	*Heterozygous*	Pathogenic	Intellectual developmental disorder-22
*KAT6A (NM_006766.5):c.1405C > T* *(p.Arg469Ter)*	*Heterozygous*	Pathogenic	Arboleda–Tham syndrome
*OTUD6B (ENST00000404789.8):c.631G > T* *(p.Glu211Ter)*	*Homozygous*	Pathogenic	Intellectual developmental disorder with dysmorphic facies, seizures, and distal limb anomalies
*TTN (NM_001267550.2):c.32471-1G > A*	*Heterozygous*	Pathogenic	Cardiomyopathy, muscular dystrophy, limb-girdle-10, myofibrillar-9 with early respiratory failure, Salih myopathy, tibial muscular dystrophy
*TRIO (ENST00000344204.9):c.2105C > A* *(p.Ser702Ter)*	*Heterozygous*	Pathogenic	Intellectual developmental disorder-44 with microcephaly, intellectual developmental disorder-63 with macrocephaly
*ATM (NM_000051.4):c.381del* *(p.Val128Ter)*	*Homozygous*	Pathogenic	Ataxia-telangiectasia, lymphoma B-cell non-Hodgkin, lymphoma mantle cell, T-cell prolymphocytic leukemia, susceptibility to breast cancer
*CDKL5 (NM_001323289.2):c.1243dup* *(p.Thr415AsnfsTer4)*	*Heterozygous*	Likely pathogenic	Developmental and epileptic encephalopathy-2
*ZBTB18 (NM_205768.3):c.32A > T* *(p.Glu11Val)*	*Heterozygous*	Likely pathogenic	Intellectual developmental disorder-22
*KDM5C (ENST00000375401.8):c.2114G > A (p.Arg705His)*	*Heterozygous*	Likely pathogenic	Intellectual developmental disorder, X-linked syndromic, Claes–Jensen type
*WWOX(NM_016373.4):c.33del (p.Asp11GlufsTer69)*	*Homozygous*	Likely pathogenic	Developmental and epileptic encephalopathy-28, esophageal squamous cell carcinoma, somatic, Spinocerebellar ataxia, autosomal recessive-12
*CACNA1G(ENST00000359106.10):c.632T > C (p.Leu211Pro)*	*Heterozygous*	Likely pathogenic	Spinocerebellar ataxia-42
*FLNA(NM_001456.4):c.7906G > A p. (Val2636Ile)*	*Hemizygous*	Novel variant^a^	Cardiac valvular dysplasia, congenital short bowel syndrome, frontometaphyseal dysplasia-1, Heterotopia periventricular-1, Intestinal pseudo-obstruction (neuronal), Melnick–Needles syndrome, otopalatodigital syndrome-I and II, terminal osseous dysplasia
*TGFBR1 (NM_004612.4):c.1433A > G* *(p.Asn478Ser)*	*Heterozygous*	VOUS	Loeys–Dietz syndrome-1, susceptibility to multiple self-healing squamous epithelioma
*ERCC1 (NM_001983.4):c.796G > A* *(p.Ala266Thr)*	*Homozygous*	VOUS	Cerebro-oculo-facioskeletal syndrome-4
*TGM1 (NM_000359.3):c.876 + 10G > A*	*Homozygous*	VOUS	Ichthyosis, congenital, autosomal recessive-1
*BRWD3 (NM_153252.5):c.3602 + 2°C > G*	*Homozygous*	VOUS	Intellectual developmental disorder-93
*USP9X (NM_001039591.3):c.90G > C (p.Gln30His)*	*Heterozygous*	VOUS	Intellectual developmental disorder-99
*WARS1 (NM_173701.2):c.317G > T* *(p.Arg106Leu)*	*Homozygous*	VOUS	Neuronopathy distal hereditary motor-IX
*PRUNE1 (NM_021222.3):c.901A > G* *(p.Ile301Val)*	*Homozygous*	VOUS	Neurodevelopmental disorder with microcephaly, hypotonia, and variable brain anomalies
*VWA8 (NM_015058.2):c.947A > G* *(p.Asp316Gly)*	*Heterozygous*	VOUS	—
*EFHC1 (NM_018100.4):c.731G > A* *(p.Arg244Gln)*	*Heterozygous*	VOUS	Susceptibility to myoclonic epilepsy-1
*KAT6B (ENST00000287239.10):c.5675C > T* *(p.Pro1892Leu)*	*Heterozygous*	VOUS	Genitopatellar syndrome, SBBYSS syndrome
*KAT6B (NM_012330.4):c.565A > T* *(p.Ser189Cys)*	*Heterozygous*	VOUS	Genitopatellar syndrome, SBBYSS syndrome
*LHX3 (NM_014564.5):c.127A > G* *(p.Ile43Val)*	*Homozygous*	VOUS	Pituitary hormone deficiency
*CLN3 (NM_001286110.2):c.754C > T* *(p.Leu252Phe)*	*Homozygous*	VOUS	Ceroid lipofuscinosis, neuronal-3
*CNTNAP2 (NM_014141.6):c.3613A > G* *(p.Ile1205Val)*	*Heterozygous*	VOUS	Pitt–Hopkins like syndrome-1, susceptibility to autism-15
*SRPX2 (NM_014467.3):c.56°C > T* *(p.Pro187Leu)*	*Homozygous*	VOUS	—
*TRIO (NM_007118.4):c.34G > T* *(p.Ala12Ser)*	*Heterozygous*	VOUS	Intellectual developmental disorder-44 with microcephaly, Intellectual developmental disorder-63 with macrocephaly
*COL6A1 (NM_001848.3):c.2614C > T* *(p.Arg872Trp)*	*Homozygous*	VOUS	Bethlem myopathy-1, Ullrich congenital muscular dystrophy 1

SNV, single-nucleotide variant; NDDs, neurodevelopmental disorders; ACMG, American College of Medical Genetics and Genomics; OMIM, Online Mendelian Inheritance in Man database; VOUS, variant of uncertain significance.

^a^ Novel variant.

**Table 3:** CNV variants found in NNDs.

Sample	Chromosome	Start	End	OMIM genes^a^	Size	Sex	Classification
9900	4 Deletion	Del (169615395 Del (180957300 Del (183714571	170822415)x1 181311082)1 188039424)x1	*PALLD, NEK1, CLCN3, TENM3, TRAPPC11, CCDC111, SLC25A4, UFSP2, TLR3, CYP4V2, KLKB1, F11*	1.2 Mb 4.32 Mb 3,542 Kb	Male	Pathogenic
79860	1 Deletion 2 Duplication	Del (449067 Dup (39896	2704774)x1 1292969)x3	*ISG15, AGRN, TNFRSF4, B3GALT6, DVL1, VWA1, ATAD3A, TMEM240, GNB1, CFAP74, GABRD, SKI, PEX10, PANK4*	2.3 Mb 1.3 Mb	Female	Pathogenic
27510	17 Duplication	Dup (15767020	20261250)x3	*ZSWIM7, TTC19, PIGL, TNFRSF13B, FLCN, RAI1, SREBF1, ATPAF2, MYO15A, MEIF2, TOP3A, GRAP, B9D1, ALDH3A2*	4.49 Mb	Male	Pathogenic
5040	15 Deletion	Del (23615768	28534245)x1	*MKRN3, MAGEL2, UBE3A, GABRB3, GABRA5, OCA2, HERC2*	4,918 Kb	Female	Pathogenic
11310	15 Duplication	Dup (22770421	28547544)x4	*NIPA1, MKRN3, MAGEL2, UBE3A, GABRB3, GABRA5, OCA2, HERC2*	5.72 Mb	Female	Pathogenic
3350	8 Duplication	Dup (7334625	11860230)x3	*RP1L1, BLK, GATA4, FDFT1*	4.522 Mb	Male	Pathogenic
98190	2 Deletion	Del (135777503	135847694)x0	*RAB3GAP1*		Male	Pathogenic
03050	2 Deletion	Del (50506323	50864204)x1	*NRXN1*	3582 kb	Female	Pathogenic
7890	3 Deletion	Del (195780280	197299811)x1	*TFRC, SLC51A, PCYT1A, DYNLT2B, RNF168, NRROS, CEP19, PAK2*	1.52 Mb	Male	Pathogenic
44660	1 Deletion	Del (2761325 Del (10264213	7422056)x1 16142227)x1	*PRDM16, TP73, SMIM1, CEP104, NPHP4, CHD5, ESPN, PLEKHG5, CAMTA1, KIF1B, PEX14, TARDBP, MASP2, MTOR, UBIAD1, MAD2L2, CLCN6, NPPA, MTHFR, PLOD1, MFN2, VPS13D, CTRC, CELA2A*	4.72 Mb 5.92 Mb	Female	Pathogenic
9630	15 Deletion	Del (23707452	28406709)x1	*MKRN3, MAGEL2, UBE3A, GABRB3, GABRA5, OCA2, HERC2*	4.72 Mb	Male	Pathogenic
86500	17 8	Dup(21529888 Dup (53214791	22261792) 53449548)	—	731.904 kb 234.7572 kb	Female	Pathogenic
38630	21 Deletion	Del (35495445	48080926)X1	*KCNE2, KCNE1, RUNX1, CLDN14, HLCS, PIGP, DYRK1A, KCNJ6, RIPK4, TMPRSS3RSPH1, WDR4, CBS, CRYAA, SIK1, HSF2BP, PDXK, CSTB, TSPEAR, TRAPPC10, AIRE, CFAP410, ITGB2, ADARB1, COL18A1, SLC19A1, COL6A2, FTCD, COL6A1, LSS, MCM3AP, PCNT*	12.58 Mb	Male	Pathogenic
6440	6 Deletion 8 Duplication	Del (168629285 Dup (129381645	170892302)x1 146280872)x3	*SMOC2, THBS2, ERMARD, DLL1, PSMB1, TBP, CCDC26, KCNQ3, LRRC6, TG, NDRG1, ZFAT1, KCNK9, TRAPPC9, AGO2, SLURP1, CYP11B1, CYP11B2, GPIHBP1, MAFA, FAM83H, PUF60, OPLAH, GPAA1, CYC1, PLEC1, DGAT1, SLC52A2, CPSF1, SLC39A4, TONSL, RECQL4*	2,263 kb 16,899 kb	Male	Pathogenic
6870	2 Deletion	Del (148934787	149048111)x1	*MBD5*	1,132 kb	Female	Likely pathogenic
64310	6 Duplication	Dup (29232208	31498036)x2∼3	*MOG, ZFP57, HLA-A, DHX16, TUBB, VARS2, CDSN, HLA-C, HLA-B*	2,2662 kb	Male	Likely pathogenic
12990	1 Deletion	Del (146535353	147824207)x1	*GLA5, GJA8*	1.32 Mb	Female	Likely pathogenic
35850	6 Duplication	Dup (45319017	45383906)x3	*RUNX2*	652 Kb	Male	VOUS
66570	8 Duplication	Dup (102914933	103234076)x3	*RRM2B*	3,192 Kb	Male	VOUS
73800	5 Duplication	Dup (178540655	178759093)x3	*ADAMTS2*	2,182 kb	Female	VOUS
2750	13 Duplication	Dup (23671134	25009594)x3	*SGCG, SACS, MIPEP*	1.32 Mb	Male	VOUS
8020	4 Duplication	Dup(90815603	91281458)x3	—	4,662 kb	Female	VOUS
021004860	7 Duplication	Dup (99661352	100491586)x3	*AP4M1, TAF6, MAP11, STAG3, TFR2, ACTL6B, GNB2, EPO, EPHB4, ACHE*	8,302 kb	Female	VOUS
028801680	3 Deletion 5 Deletion	Del (16255442 Del (66110530	16355656)x1 66268829) x1	—	1,002 kb 1,582 kb	Female	VOUS
028076610	1 Deletion	Del (79344928	79427515)x1	—	832 kb	Female	VOUS
020311940	2 Duplication	Dup (96468158	96809264)x3	*ASTL*	341.1062 kb	Female	VOUS

NDDs, neurodevelopmental disorders; CNVs, copy number variations; VOUS, variant of uncertain significance.

^a^ Gene(s) located within the specified locus and listed in the Online Mendelian Inheritance in Man (OMIM) database as potentially contributing to a disease (according to the University of California Santa Cruz Genome Browser, GRCh37/hg19).

## 4. Discussion

**Figure 3:**
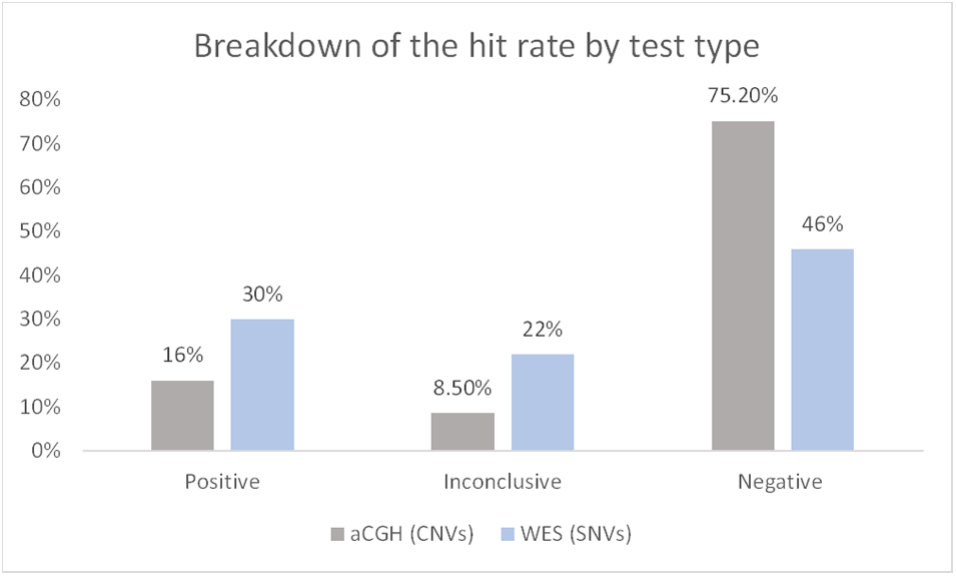
Breakdown of the hit rate by test type. The number of solved cases (CNVs/SNVs) was divided by the total number of cases. Data represented as percentage. CNVs, copy number variations; SNVs, single-nucleotide variants.

Our study describes the diagnostic yield of WES and CGH in 105 pediatric patients diagnosed with NDDs, which include developmental delay disorder, epilepsy, intellectual disability, and learning disorder. We performed an analysis of data from the reports available in electronic health records at KASCH. We found that the diagnostic yield of WES was higher at 30% compared to CGH testing (16%) (Figure 3).

WES studies have reported varying levels of diagnostic success (23–25). A study (26) found a diagnostic yield of approximately 25% when used in pediatric populations with NDDs. In a meta-analysis, Srivastava et al. (27) reported that the overall diagnostic yield of WES was 36%, with 31% for isolated NDDs and 53% for NDDs accompanied by additional conditions, outperforming microarray analysis. This is consistent with our findings. It should be noted that these diagnostic yields can be influenced by patients’ phenotypes and the population being tested.

WES revealed the presence of several genes linked with development delays, such as *VPS13B*, *RNASEH2A*, and *WWOX*. Moreover, a novel variant found by WES in the *FLNA* gene was implicated in developmental delay. *De novo* SNVs were identified by WES in two genes involved in development delay and intellectual disability in one patient, the *ZBTB18* variant *c.139°C > T* in heterozygous form was identified and classified as pathogenic. The variant analysis revealed a missense disease-causing variant in the Zn3 domain of the ZBTB18 protein. This variant was reported previously in a patient with severe intellectual disability. However, despite the cognitive impairment, the patient could live with minimal supervision, and the electroencephalogram was normal (28). Another variant found by WES was the *c.1139C > T* (p.Thr380Met) in the *PAH* gene, as a homozygous variant and classified as pathogenic. This variant is a missense variant affecting the splicing of the *PAH* gene that was reported to cause a deficiency in the activity of the phenylalanine hydroxylase. The heterozygous variant reduced the activity of the PAH enzyme by 38% (29). As a result, the patient’s homozygous variants resulted in phenylketonuria (PKU), an inborn error of metabolism that caused the severe developmental delay in the patient. Another example of SNVs found in this study is the heterozygous variant *c.493G > T* in the *TCF12* gene, which was classified as pathogenic. We also found SNVs in a male patient with intellectual disability in a gene (*TRIO*) with *c.2105C > A* variant in heterozygous form and classified as pathogenic. This indicates that mutations of *TRIO* gene are not restricted to the Caucasian population and underlie NDDs in the Middle Eastern population as well. Different mutations have been reported previously in the Caucasian population, emphasizing the *TRIO* gene's role in NDDs (30). This gene plays a fundamental role in mammalian neuronal development. It is a member of the Dbl family that encodes a guanine nucleotide exchange factor (GEF) that facilitates the activation of Rho GTPases such as RAC1, which in turn controls actin cytoskeleton dynamics.

In this study we found the diagnostic yield of CGH to be 16%. An example of CNVs found in this study is a male patient with an apparently *de novo* complex interstitial rearrangement of the long arm of chromosome 4, including a deletion of at least 1.2 Mb extending from cytogenetic band 4q32.3 to 4q33 as well as deletion of at least 4.3 Mb extending from cytogenetic band 4q35.1 to 4q35.2. He also has a deletion of 354 kb between those regions in band 4q34.3. This individual was diagnosed with developmental delay, learning disability, and speech delay. Moreover, *de novo* terminal deletion of at least 2.3 Mb was found in a female patient extending from cytogenetic band 1p36.33 to 1p36.32 and an apparently *de novo* terminal duplication of at least 1.3 Mb within cytogenetic band 2p25.2. This individual was diagnosed with developmental delay. Another example of CNVs found in this study is a terminal deletion of at least 2.3 Mb within cytogenetic band 6q27 and a terminal duplication of at least 16.9 Mb extending from cytogenetic band 8q24.21 to 8q24.3 found in a male patient diagnosed with developmental delay, microcephaly, dysmorphic features, intellectual disability, and seizure. The total reported VOUS and/or possible benign variants in this study were 25% (27/105) between 18 SNVs and 9 CNVs. An example of SNVs found in this study as a VOUS is a heterozygous variant *c.5675C > T* in gene *KAT6B* found in a male patient diagnosed with developmental delay and intellectual disability. We found CNVs one copy gain within 5q35.3 region on long arm of chromosome 5 as VOUS in intellectual disorder patients.

This result sheds light on challenges faced during molecular diagnosis among NDD patients, which could be ascribed to the extensive phenotypic similarity shared among NDD patients. Moreover, mutations in several genes could share same phenotypes. Therefore, the diagnostic yield of ∼30% considers a good benchmark for successful resolution of molecular diagnosis in NDDs. It is highly recommended to create an ethnic-specific panel for NDDs, until then it is valuable to record and document all the genetic variations and phenotypes associated with developmental delays to accelerate the detection process (31).

In summary, our study demonstrates the usefulness of the high diagnostic yield by WES coupled with its role in elucidating unusual genetic mechanisms and revealing the presence of several genes linked with NDDs. Despite these advantages, there are some limitations. WES has certain limitations in detecting certain genetic variations, such as large insertions/deletions, chromosomal rearrangements, and mutations in regulatory regions. The retrospective design of this study precluded the ability to find karyotype reports for structural abnormalities on all of our patients with NDDs, which may have limited our understanding of the chromosomal rearrangements present in these patients. Additionally, variants located in genes with unknown functions may be excluded from clinical WES analysis. Furthermore, the complexity of interactions between genes and environmental factors in the development of NDDs remains an area of ongoing research and was not examined in this study. Some of these limitations may explain why 37 patients remained undiagnosed even after WES analysis. Taking into account all of these limitations, this study suggests that WES was a better approach than CGH, and these findings could help clinicians, researchers, and testing laboratories select the most cost-effective and appropriate approach for their patients.

## Data Availability

The data presented in the study are deposited in the ClinVar database repository, accession number SCV003803002- SCV003803013. SUB12655703-Review & Submit I ClinVar File Submission I Submission Portal (nih.gov)

## References

[1] Burack JA. The development of autism: perspectives from theory and research. Montreal, Canada: Lawrence Erlbaum (2001).

[2] Fazzi E Emilio B. Visual impairments and neurodevelopmental disorders; rehabilitation. Montrouge, France (2016).

[3] Quintela I Eiris J Gomez-Lado C Perez-Gay L Dacruz D Cruz R Copy number variation analysis of patients with intellectual disability from north-west Spain. Gene. (2017) 626:189–99. 10.1016/j.gene.2017.05.032 28506748

[4] Vlaskamp DRM Callenbach PMC Rump P Giannini LAA Dijkhuizen T Brouwer OF Copy number variation in a hospital-based cohort of children with epilepsy. Epilepsia Open. (2017) 2(2):244–54. 10.1002/epi4.12057 29588953 PMC5719854

[5] Thomas MSC Annette K-S. Neurodevelopmental disorders (2014).

[6] Li JJ. Multi-method investigation of gene-environment interplay and ADHD. Los Angeles, CA: University of California, Los Angeles (2013). UCLA. ProQuest ID: Li_ucla_0031D_11216. Merritt ID: ark:/13030/m54x6nsc. Retrieved from https://escholarship.org/uc/item/0cz3t955

[7] Tager-Flusberg H. Neurodevelopmental disorders. Cambridge, MA: MIT Press (1999).

[8] McGrath LM Yu D Marshall C Davis LK Thiruvahindrapuram B Li B Copy number variation in obsessive-compulsive disorder and Tourette syndrome: a cross-disorder study. J Am Acad Child Adolesc Psychiatry. (2014) 53(8):910–9. 10.1016/j.jaac.2014.04.022 25062598 PMC4218748

[9] Michel J-M. Understanding autism: parents, doctors and the history of a disorder, by Chloe Silverman, Princeton, NJ, Princeton University Press, 360 pp., 2011, $35.00/£24.95 (paperback), ISBN 978-0-69-115046-8. Disabil Soc. (2012) 27(7):1039–41. 10.1080/09687599.2012.722411

[10] Gall J Nizon M Beneteau F Cormier-Daire C Ferec M Gilbert-Dussardier S Sex chromosome aneuploidies and copy-number variants: a further explanation for neurodevelopmental prognosis variability? Eur J Hum Genet. (2017) 25(8):930–4. 10.1038/ejhg.2017.93 28612834 PMC5567159

[11] Retz W. Attention deficit hyperactivity disorder in adults. Basel: S Karger AG (2010).

[12] Al-Qahtani M Kalamegam G Jan M Assidi M Naseer M Ansani S Copy number variations in Saudi family with intellectual disability and epilepsy. BMC Genom. (2016) 17:61–9. 10.1186/s12864-015-2291-9 PMC5073808 27766957

[13] Scherer S. Copy number variation. London: Henry Stewart Talks (2009).

[14] Naseer MI Faheem M Chaudhary AG Kumosani TA Al-Quaiti MM Jan MM Genome wide analysis of novel copy number variations duplications/deletions of different epileptic patients in Saudi Arabia. BMC Genom. (2015) 16(Suppl 1):S10. 10.1186/1471-2164-16-S1-S10 PMC4315149 25923336

[15] Kehrer-Sawatzki CDNA. Copy number variation and disease: 51 tables. Basel: S Karger AG (2009).

[16] Dumas L Kim YH Karimpour-Fard A Cox M Hopkins J Pollack JR Gene copy number variation spanning 60 million years of human and primate evolution. Genome Res. (2007) 17(9):1266–77. 10.1101/gr.6557307 17666543 PMC1950895

[17] Kariminejad R. Copy number variations in structural brain malformations. Berlin-Dahlem: Freie Universitat (2012).

[18] Naseer MI Chaudhary AG Rasool M Kalamegam G Ashgan FT Assidi M Copy number variations in Saudi family with intellectual disability and epilepsy. BMC Genom. (2016) 17(Suppl 9):757. 10.1186/s12864-016-3091-6 PMC5073808 27766957

[19] Mitchell KJ. The genetics of neurodevelopmental disorders. *Curr Opin Neurobiol*. (2011) 21(1):197–203. 10.1016/j.conb.2010.08.009 20832285

[20] Beaudet AL. The utility of chromosomal microarray analysis in developmental and behavioral pediatrics. Dublin: Childhood Development Initiative (2013). 10.1111/cdev.12050 PMC3725967 23311723

[21] Kaminsky EB Kaul V Paschall J Church DM Bunke B Kunig D An evidence-based approach to establish the functional and clinical significance of copy number variants in intellectual and developmental disabilities. Genet Med. (2011) 13(9):777–84. 10.1097/GIM.0b013e31822c79f9 21844811 PMC3661946

[22] Richards S Aziz N Bale S Bick D Das S Gastier-Foster J Standards and guidelines for the interpretation of sequence variants: a joint consensus recommendation of the American college of medical genetics and genomics and the association for molecular pathology. Genet Med. (2015) 17(5):405–24. 10.1038/gim.2015.30 25741868 PMC4544753

[23] Leite A Pinto IP Leijsten N Ruiterkamp-Versteeg M Pfundt R de Leeuw N Diagnostic yield of patients with undiagnosed intellectual disability, global developmental delay and multiples congenital anomalies using karyotype, microarray analysis, whole exome sequencing from central Brazil. PLoS One. (2022) 17(4):e0266493. 10.1371/026649 35390071 PMC8989190

[24] Clark MM Stark Z Farnaes L Tan T White SM Dimmock D Meta-analysis of the diagnostic and clinical utility of genome and exome sequencing and chromosomal microarray in children with suspected genetic diseases. NPJ Genom Med. (2018) 3:16. 10.1038/s41525-018-0053-8 30002876 PMC6037748

[25] Yang Y Muzny D Reid J Bainbridge M Willis A Ward P Clinical whole-exome sequencing for the diagnosis of Mendelian disorders. N Engl J Med. (2013) 369:1502–11. 10.1056/NEJMoa1306555 24088041 PMC4211433

[26] Dharmadhikari AV Ghosh R Yuan B Liu P Dai H Al Masri S Copy number variant and runs of homozygosity detection by microarrays enabled more precise molecular diagnoses in 11,020 clinical exome cases. Genome Med. (2019) 11(1):30. 10.1186/s13073-019-0639-5 31101064 PMC6525387

[27] Srivastava S Love-Nichols JA Dies KA Ledbetter DH Martin CL Chung WK Meta-analysis and multidisciplinary consensus statement: exome sequencing is a first-tier clinical diagnostic test for individuals with neurodevelopmental disorders. Genet Med. (2019) 21(11):2413–21. 10.1038/s41436-019-0554-6 31182824 PMC6831729

[28] Cohen JS Srivastava S Farwell Hagman KD Shinde DN Huether R Darcy D Further evidence that de novo missense and truncating variants in ZBTB18 cause intellectual disability with variable features. Clin Genet. (2017) 91(5):697–707. 10.1111/cge.12861 27598823

[29] Heintz C Dobrowolski SF Andersen HS Demirkol M Blau N Andresen BS. Splicing of phenylalanine hydroxylase (PAH) exon 11 is vulnerable: molecular pathology of mutations in PAH exon 11. Mol Genet Metab. (2012) 106(4):403–11. 10.1016/j.ymgme.2012.05.013 22698810

[30] Pengelly RJ Heygate SG Schmidt S Seaby EG Jabalameli R Methta SG Mutations specific to the Rac-GEF domain of TRIO cause intellectual disability and microcephaly. J Med Genet. (2016) 53:735–42. 10.1136/jmedgenet-2016-103942 27418539 PMC5264232

[31] Jabalamelil RM. Diagnostic outcomes of exome gene panel sequencing in patients with unusual syndromic cleft lip/palate phenotypes. *BioRxiv*. (2018).

