# Supplementary material for: The diagnostic yield of CGH and WES in neurodevelopmental disorders
Source: Front Pediatr. 2023 Mar 1;11:1133789. doi: 10.3389/fped.2023.1133789 (PMC10014736; doi:10.3389/fped.2023.1133789)
Supplement: Supplementary file 1 [file Datasheet1.pdf]

Table 4 Reported variants; rs ID variant number, location G37, the pathogenicity score of the variants, Sorting Intolerant from Tolerant (SIFT), conservation score Genomic Evolutionary Rate Profiling (GERP), Combined Annotation Dependent Depletion (CADD), Deciphering Developmental Disorders (DDD).

SNVs	Location G37	RS number	SIFT	GERP	CADD	AF (gnomAD)	DDD consortium
VPS13B(NM_152564.5):c.1219C>T (p.Gln407Ter)	*8:100146872*	*rs386834070*	*NA*	*5.25*	*39*	*NA*	*NA*
RNASEH2A(NM_006397.3):c.557G>A (p.Arg186Gln)	*19:12921138*	*rs753679297*	*Pathogenic*	5.3099	27.7	*3.98E-06*	*NA*
WWOX(NM_016373.4):c.606-1G>A	*16:78458766*	*rs730882215*	*NA*	*5.3499*	*34*	*NA*	*NA*
PAH(NM_001354304.2):c.1139C>T (p.Thr380Met)	*12:103237484*	rs62642937	*Pathogenic*	*5.34*	*24.2*	*3.19E-05*	*NA*
PTPN11(NM_001330437.2):c.1519G>A (p.Gly507Arg)	*12:112926887*	*rs397507545*	*Pathogenic*	5.13	31	*NA*	*NA*
SPAST(NM_014946.4):c.1496G>A (p.Arg499His)	*2:32366975*	rs878854991	*Pathogenic*	*4.98*	*31*	*NA*	*NA*
MYT1L(NM_001329851.2):c.1585G>A (p.Gly529Arg)	*2:1921010*	*rs1275489527*	*NA*	*5.73*	*32*	*NA*	*NA*
TBCD(NM_005993.5):c.1661C>T (p.Ala554Val)	*17:80858538*	rs1555641324	*Uncertain*	5.23	24.5	*NA*	*NA*
SLC19A3(NM_025243.4):c.1264A>G (p.Thr422Ala)	*2:228552932*	rs121917884	*Pathogenic*	5.34	24.8	*NA*	*NA*
TCF12(NM_001322164.2):c.493G>T (p.Gly165Trp)	*15:57484422*	*NA*	*Pathogenic*	5.57	28.2	*NA*	*NA*
QARS1(NM_005051.3):c.1058G>T (p.Gly353Val)	*3:49137729*	rs752600100	*Pathogenic*	5.21	24.1	*NA*	*NA*
MCOLN1(NM_020533.3):c.1336G>A (p.Val446Met)	*19:7594575*	rs754097561	*Pathogenic*	5.4699	26.1	*7.95E-06*	*NA*
BRAF(NM_001378474.1):c.1729C>A	*7:140453999*	*NA*	*NA*	5.26	27.6	*NA*	*NA*

Table 4 Reported variants; rs ID variant number, location G37, the pathogenicity score of the variants, Sorting Intolerant from Tolerant (SIFT), conservation score Genomic Evolutionary Rate Profiling (GERP), Combined Annotation Dependent Depletion (CADD), Deciphering Developmental Disorders (DDD).

(p.Leu577Ile)							
ZBTB18(NM_205768.3):c.1390C>T<br>(p.Arg464Cys)	*1:244218466*	*rs750922282*	*Pathogenic*	*5.78*	*32*	*NA*	*1*
KAT6A(NM_006766.5):c.1405C>T<br>(p.Arg469Ter)	*8:41832299*	*NA*	*NA*	*5.6799*	*36*		*1*
OTUD6B(ENST00000404789.8):c.631G>T<br>(p.Glu211Ter)	*8:92092899*	rs772032719	*NA*	*5.84*	*40*	*NA*	*NA*
TTN(NM_001267550.2):c.32471-1G>A	*2:179549717*	*rs371725574*	*NA*	*5.8099*	*35*	*1.36E-05*	*NA*
TRIO(ENST00000344204.9):c.2105C>A<br>(p.Ser702Ter)	*5:14358345*	*NA*	*NA*	*4.61*	*41*	*3.19E-05*	*NA*
ATM(NM_000051.4):c.381del<br>(p.Val128Ter)	*11:108106446*	*rs587781831*	*NA*	*-1.22*		*1.59E-05*	*NA*
CDKL5(NM_001323289.2):c.1243dup<br>(p.Thr415AsnfsTer4)	*X:18622283*	*NA*	*NA*	*NA*		*NA*	*NA*
ZBTB18(NM_205768.3):c.32A>T<br>(p.Glu11Val)	*1:244217108*	*NA*	*Pathogenic*	*5.2199*	*29.2*	*NA*	*NA*
KDM5C(ENST00000375401.8):c.2114G>A<br>(p.Arg705His)	*X:53228288*	*rs1569264240*	*Pathogenic*	*4.69*	*28.1*	*NA*	*NA*
WWOX(NM_016373.4):c.33del<br>(p.Asp11GlufsTer69)	*16:78133708*	*NA*	*NA*	*5.11*		*NA*	*NA*
CACNA1G(ENST00000359106.10):c.632T>C<br>(p.Leu211Pro)	*17:48649284*	*NA*	*Pathogenic*	*4.4499*	*26.8*	*NA*	*NA*
FLNA(NM_001456.4):c.7906G>A p.(Val2636Ile)	*x:153577231*	*rs1557175101*	*Benign*	*1.45*	*14.55*	*5.69E-06*	*NA*

Table 4 Reported variants; rs ID variant number, location G37, the pathogenicity score of the variants, Sorting Intolerant from Tolerant (SIFT), conservation score Genomic Evolutionary Rate Profiling (GERP), Combined Annotation Dependent Depletion (CADD), Deciphering Developmental Disorders (DDD).

TGFBR1(NM_004612.4):c.1433A>G (p.Asn478Ser)	*9:101911508*	*rs141259922*	*Benign*	*5.6599*	*22.7*	*2.73E-04*	*NA*
ERCC1(NM_001983.4):c.796G>A (p.Ala266Thr)	*19:45916982*	rs3212977	*Uncertain*	*4.98*	*25*	*4.92E-04*	*NA*
TGM1(NM_000359.3):c.876+10G>A	*14:24729136*	*rs201936986*	*NA*	*-0.4539*	*3.12*	*NA*	*NA*
BRWD3(NM_153252.5):c.3602+20C>G	*X:79946532*	*rs1602309498*	*NA*	*0.4629*	*0.8*	*NA*	*NA*
USP9X(NM_001039591.3):c.90G>C(p.Gln30His)	*X:40982971*	*rs754357906*	*Benign*	*4.5399*	*21.5*	*1.68E-05*	*NA*
WARS1(NM_173701.2):c.317G>T (p.Arg106Leu)	*14:100826996*	*NA*	*Pathogenic*	*4.51*	*26.6*	*NA*	*NA*
PRUNE1(NM_021222.3):c.901A>G (p.Ile301Val)	*1:151001388*	*rs1558088029*	*Benign*	*-4.98*	*9.15*	*NA*	*NA*
VWA8(NM_015058.2):c.947A>G (p.Asp316Gly)	*13:42460084*	*rs549867896*	*Uncertain*	*5.5599*	*24.4*	*2.08E-05*	*NA*
EFHC1(NM_018100.4):c.731G>A (p.Arg244Gln)	*6:52318900*	*rs140476054*	*Uncertain*	*5.8699*	*31*	*8.84E-05*	*NA*
KAT6B(ENST00000287239.10):c.5675C>T (p.Pro1892Leu)	*10:76790257*	*rs1037774698*	*Uncertain*	*5.5399*	*26.4*	*NA*	*NA*
KAT6B(NM_012330.4):c.565A>T (p.Ser189Cys)	*10:76603180*	*NA*	*Uncertain*	*4.92*	*22.6*	*NA*	*NA*
LHX3(NM_014564.5):c.127A>G (p.Ile43Val)	*9:139092567*	*rs1341469393*	*Pathogenic*	*3.48*	*22.1*	*3.99E-06*	*NA*
CLN3(NM_001286110.2):c.754C>T (p.Leu252Phe)	*16:28493694*	*rs774239875*	Uncertain	*4.55*	*24*	*7.97E-06*	*NA*

Table 4 Reported variants; rs ID variant number, location G37, the pathogenicity score of the variants, Sorting Intolerant from Tolerant (SIFT), conservation score Genomic Evolutionary Rate Profiling (GERP), Combined Annotation Dependent Depletion (CADD), Deciphering Developmental Disorders (DDD).

CNTNAP2(NM_014141.6):c.3613A>G (p.Ile1205Val)	*7:148080878*	*NA*	*Benign*	*-0.6949*	*10*	*NA*	*NA*
SRPX2(NM_014467.3):c.560C>T (p.Pro187Leu)	*X:99920267*	*rs1569361725*	*Pathogenic*	*5.61*	*28.6*	*NA*	*NA*
TRIO(NM_007118.4):c.34G>T (p.Ala12Ser)	*5:14143868*	*NA*	*Pathogenic*	1.2699	11.93	*NA*	*NA*
COL6A1(NM_001848.3):c.2614C>T (p.Arg872Trp)	*21:47423454*	*rs368561027*	*Pathogenic*	*3.94*	*24.3*	*1.05E-04*	*NA*
